# Recent Progress in Advanced Materials for Lithium Ion Batteries

**DOI:** 10.3390/ma6010156

**Published:** 2013-01-10

**Authors:** Jiajun Chen

**Affiliations:** Toyota Research Institute of North America, 1555 Woodridge Avenue, Ann Arbor, MI 48105, USA; E-Mails: jiajunchen03@yahoo.com or jack.chen@tema.toyota.com; Tel.: +1-734-995-5313.

**Keywords:** lithium-ion battery, anodes, cathodes, synthesis

## Abstract

The development and commercialization of lithium ion batteries is rooted in material discovery. Promising new materials with high energy density are required for achieving the goal toward alternative forms of transportation. Over the past decade, significant progress and effort has been made in developing the new generation of Li-ion battery materials. In the review, I will focus on the recent advance of tin- and silicon-based anode materials. Additionally, new polyoxyanion cathodes, such as phosphates and silicates as cathode materials, will also be discussed.

## 1. Introduction

The inevitable depletion of non-renewable fossil fuels and environmental issues, such as CO_2_ emissions, force us to transit away from using fossil fuels as the main global energy source. Green energy sources, such as solar, hydroelectric, thermal and wind energy capture will eventually replace traditional energy sources. Most of these renewable energy sources, such as solar and wind, are typically periodic or intermittent. Electrical energy storage, such as batteries, is crucial to solve the problem, as they can efficiently store electricity in chemicals and release it according to demand.

Current lithium ion battery technology offers the highest energy density among the rechargeable battery technologies, dominating the market for mobile electronic devices for decades. However, alternative forms of transportation, such as plug-in hybrid electric vehicles (PHEV) and all electric vehicles (EV), require significant improvements in many perspectives, such as energy density, safety, durability and cost. The key to the success of development of novel and advanced rechargeable batteries are the materials. Demonstrated by Whittingham *et al.*, TiS_2_ was shown to serve as a host for reversible intercalated and de-intercalated lithium into its structure [1]. The single phase behavior during cycling enables it to fully remove and insert lithium ions reversibly. In the 1970s, the discovery of TiS_2_ resulted in Exxon’s cells as large as 45 Wh. In the original Exxon LiTiS_2_ batteries, pure lithium served as the anode. The dendritic growth of lithium metal during cycling can short out the cell, which will potentially lead to explosion hazards [2]. In the 1990s, carbonaceous material was discovered as a highly reversible and low voltage Li intercalation–deintercalation anode, as it possesses unique properties. Combining safety features of the carbon anode and the high voltage LiCoO_2_ cathode, SONY commercialized the lithium ion battery (C/LiCoO_2_ cell), which now dominates the market for electronics. Although carbon-based anodes have the advantage of a long cycle life, low cost and abundance, the graphite anode has significant disadvantages with regards to low gravimetric and volumetric specific capacity (372 mAh/g and 833 mAh/cm^3^). Lithium–metal alloys, such as lithium–tin, are among the most promising materials to replace current carbon-based anodes, because of their high capacity. The recent commercial success of these anodes includes nanostructured SnCo anodes used in SONY’s Nexelion battery, which has a 50% increase in volumetric capacity over the conventional battery [3].

The key requirements and criteria for a material to be successfully used as a cathode or anode in a lithium ion battery have been discussed in several excellent comprehensive reviews to which the reader is referred for details [2,4]. Progress describing the patents awarded on synthesizing nanostructured Li-ion cathode material, with the focus on low temperature synthesis (especially hydrothermal synthesis), has recently been reviewed [5]. This article aims to provide a useful survey of the most recent progress on the development of Li-ion battery materials. To begin, a brief review of new polyoxyanion compounds, such as phosphates and silicates as cathode materials, is given, and the rest of the sections will be focused on the recent advance of tin- and silicon-based anode materials.

## 2. Cathodes

### 2.1. Olivine-Structured LiMPO_4_ (M = Fe, Mn, Co, Ni)

Inspired by the seminal work of Goodenough and coworkers, LiFePO_4_ has been the focus of research in developing environmentally friendly, low cost and high performance cathode materials for lithium ion batteries [6]. The discharge potential of LiFePO_4_ is around 3.45 V *versus* (Li/Li^+^), and the theoretical capacity is 170 mAh/g. Thus, the gravimetric energy density of LiFePO_4_ is about 586 Wh/kg, which is slightly higher than LiCoO_2_. Initial reports on the electrochemical characteristics of LiFePO_4_ emphasized its low capacity and poor rate capability due to its extremely low ionic and electronic conductivity. Pure LiFePO_4_ behaves as an insulator, which has conductivity as low as ~10^−9^ S/cm. Significant work has been done to improve its electrochemical properties [7,8,9,10,11]. It was found that carbon coating can effectively enhance the conductivity of the LiFePO_4_ cathode composite [12]. As long as particle sizes were kept small and particle size distributions were narrow, enhanced electrochemical performance was observed. Reduced Li-ion diffusion distances and carbon coating ameliorates the effects of low electronic conductivity, allowing full charge and discharge at high rates.

LiFePO_4_ is less dense than the layered oxides or spinels (the crystallographic density is 3.6 g/cm^3^), thus the volumetric energy density is about 2100 Wh/l, which is slightly lower than LiCoO_2_. However, its low cost, long life and environmental friendliness makes it ideal for the next generation cathode materials. To take advantage of the low cost of raw materials, low cost syntheses are highly desirable. Among various synthetic techniques, hydrothermal synthesis confers unique advantages, *i.e.*, low cost, easy to scale up and a good control over the product’s purity and crystallinity. As summarized in a previous review [5], 10 years of research has resulted in the successful commercialization of hydrothermal processes for manufacturing LiFePO_4_, although improvements are needed to further lower the manufacturing cost by eliminating the additional post-heat treatment process.

**Figure 1:**
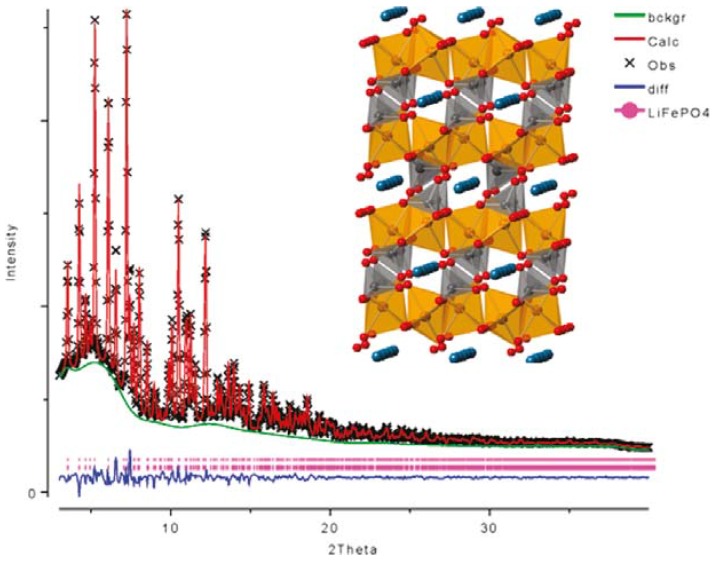
High-resolution synchrotron X-ray diffraction (XRD) and Rietveld refinement patterns from hydrothermally prepared LiFePO_4_. Adapted with permission from [16]. Copyright 2011 American Chemical Society.

The defects are of great interest in this material; namely, anti-site defects in the olivine structure. Li-ion can diffuse quite fast in the 1D channels of the olivine structure, however those channels are easily blocked by defects. Previous experimental studies showed that the presence of larger size transition metal cations, which are much less mobile, impede the fast diffusion of Li-ion. LiFePO_4_ synthesized at low temperature (120 °C) demonstrated almost 7% iron occupancy on the lithium sites. As a consequence, the electrochemical capacity of these highly defected materials was quite small, even at low current densities [13]. To further understand and confirm the defects in the structure of LiFePO_4_, growing single crystals is essential. Chen *et al.* synthesized millimeter-sized LiFePO_4_ single crystals via the hydrothermal method [14]. The colorless to light green single crystals were formed with the assistance of a large quantity of ascorbic acid and polyethylene glycol. The crystal structure of (Li_0.938_Fe_0.031_)FePO_4_ was determined by X-ray single crystal diffraction analysis. Excess Fe ions on Li sites were accompanied by a small number of vacancies. The general formula [Li_1 − 2*x*_Fe*_x_*]FePO_4_ is concluded as the best structural solution. Recent *ab initio* calculations demonstrate that a point defect of even less than 1% can tremendously limit Li-conductivity [15]. Computational results showed a decrease in the diffusion coefficient for Li transport by more than two orders of magnitude, with defect concentrations as small as 0.5%. Time-resolved, synchrotron X-ray diffraction is a powerful tool for understanding the defect formation or elimination process [16,17]. Figure 1 shows high-resolution synchrotron XRD and Rietveld refinement patterns from hydrothermally prepared LiFePO_4_.

**Figure 2:**
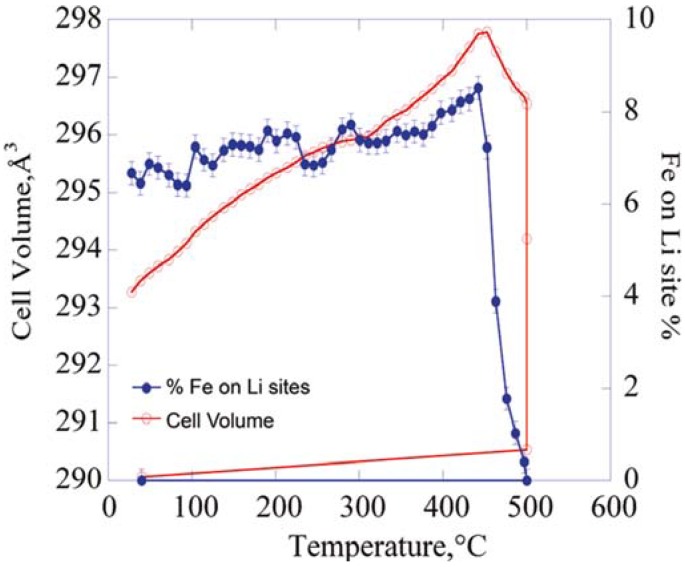
Unit cell volume and the concentration (percent) of iron on lithium sites as a function of post-synthesis heating temperature. Adapted with permission from [16]. Copyright 2011 American Chemical Society.

The recent real-time measurement confirmed that although significant amounts of defects could exist in the LiFePO_4_ at room temperature, these anti-site defects can be completely eliminated above 500 °C, as shown in Figure 2 Defect-free material obtained by post-heat treatment at 500 °C shows specific capacity increasing by approximately 60% at a C/20 rate [16]. The results suggested that the electrochemical performance may be significantly enhanced by milder post-synthesis heating. Very recently, Recham *et al.* also demonstrated that defect-free LiFePO_4_ single crystals of 700 μm size can be formed if the hydrothermal reaction holds at 260 °C for a month in water-ionic liquid media, and smaller ones (80–120 μm) can be synthesized in pure water [18]. After undergoing ball milling and carbon coating, the single crystal LiFePO_4_ samples were electrochemically active. At the elevated temperature of 100 °C, it had a reversible capacity of 145 mAh/g with excellent retention and less than 5% fading after 100 cycles. Large size LiMnPO_4_ and LiCoPO_4_ single crystals were also obtained by this approach.

The discharge potential of the iso-structural LiMnPO_4_ is about 0.5 V *vs.* Li/Li^+^ higher than that of LiFePO_4_, resulting in a 15% increase in the theoretical gravimetric energy density. LiMnPO_4_ suffers from much lower intrinsic electrical and ionic conductivities than LiFePO_4_. Carbon coating, reduction of particle size to the nanoscale and partial substitution of Mn with Mg or Fe has been extensively studied to enhance the performance of LiMnPO_4_ [19,20].

Recently, Oh *et al.* synthesized carbon-LiMnPO_4_ nano-composite by ultrasonic spray pyrolysis and ball milling [21]. The nano-composite contained 30% acetylene black (AB) carbon, which can deliver discharge capacities of 155 mAh/g and 107 mAh/g at 0.1 C and 2 C, respectively. The capacity retention of the 30 wt % AB composite is quite good at 25 °C and 55 °C. Initial capacity of 94.2% at 25 °C and 87.7% at 55 °C were maintained after 50 cycles. Oh *et al.* contributed the enhanced performance to the homogeneous coating of the acetylene black carbon, which can protect C-LiMnPO_4_ against electrolyte (HF) attack during extensive cycling.

More recently, Yoo *et al.* designed 3D microporous (3DM) structure LiMnPO_4_ by using a polymethylmethacrylate (PMMA) template [22]. After calcination, the PMMA template was removed and LiMnPO_4_ nanoparticles were well dispersed in the carbon matrix. 3DM balls and flakes can be obtained by this method. The 3DM structure combined several advantages, such as reduction of solid state diffusion length of lithium ions, increased the surface area and enhanced electronic conductivity in carbon composite. Thus, significantly improved performance was observed. The electrochemical tests were carried out at a 0.1 C rate to charge the cell to 4.6 V, which is held for 2 h before discharging. The first discharge capacity for 3DM balls at the 0.1 C rate is 162 mAhg, with no significant capacity fading observed up to 50 cycles. The rate performance is also much better than those LiMnPO_4_ nanoparticles without 3DM structure.

The high potential of LiCoPO_4_ and LiNiPO_4_ (4.8 V and 5.1 V *vs.* Li/Li^+^, respectively) makes them much less studied due to a lack of the stable electrolyte in the voltage window. The operating voltage around 5 V causes significant side reactions to occur at the LiCoPO_4_ electrode/electrolyte interface. Strategies, such as carbon coating, are necessary for both enhancing the kinetics of LiCoPO_4_ cathode and preventing the electrode/electrolyte interfacial side reaction. Recently, Ni *et al.* prepared LiCoPO_4_/C Core/Shell composite by the sol-gel method [23]. In the voltage range of 3.0–5.2 V, a specific capacity of 131 mAh/g can be achieved during the first cycle. At a low current of 17 mA/g, 78% of initial capacity was maintained after 40 cycles. Oh *et al.* developed a planetary ball milling method to make LiCoPO_4_–acetylene black carbon composites [24]. Discharge capacity of 145 mAh/g was obtained at a rate of C/10 and a reversible capacity of 100 mAh/g can be maintained over 50 cycles. Both of these composites show quite good capacity retention compared to pure LiCoPO_4_ cathode. This can be attributed to the homogeneous carbon coating or shell on the surface, which can suppress side reactions.

Interestingly, Sharabi *et al.* showed switching from conventional polyethylene (PE) separators to SiO_2_-containing separators can greatly improve capacity retention of LiCoPO_4_ [25]. Specific capacity of 100 mAh/g can be maintained over the 200 cycles. The structural degradation of the LiCoPO_4_ cathode during cycling can be minimized by using quartz (SiO_2_) separators.

### 2.2. Orthosilicates Li_2_MSiO_4_ (M = Fe, Mn, Co)

More recently, Orthosilicates Li_2_MSiO_4_(M = Fe, Mn, Co) are receiving considerable attention as a new class of polyoxyanion cathodes for Li-ion battery. Li_2_MSiO_4_ provides the potential of insertion/extraction of two lithium ions per formula unit. This corresponds to a high theoretical capacity of about 330 mAh/g, which is almost twice the capacity of olivine lithium metal phosphate materials. One of the most attractive materials in this family is Li_2_FeSiO_4_, because iron and silicon are among the most abundant and cheapest elements. The major drawback of the silicate family is their intrinsic low electronic conductivity (5 × 10^−16^ S/cm for L_i2_MnSiO_4_ and 6 × 10^−14^ S/cm for Li_2_FeSiO_4)_, which has been shown to be up to several orders of magnitude lower than that of LiMnPO_4_ or LiFePO_4_ [26].

Li_2_FeSiO_4_ orthorhombic structure is related to the Li_3_PO_4_ structure with space group Pmn2_1_. Several polymorphs are also available and exist in two main classes, *β* and *γ* phases. Nyten *et al.* reported that *β-*phase Li_2_FeSiO_4_ (based on *β*-Li_3_PO_4_) can be synthesized by solid-state reaction [27]. The refined lattice parameters for Li_2_FeSiO_4_ were a = 6.2661(5) Å, b = 5.3295(5) Å and c = 5.0148(4) Å. In order to increase the ionic transportation (kinetics) of as-synthesized material, the electrochemical test was conducted at an elevated temperature (60 °C). The electrochemical behavior of this material showed the capability to remove one lithium ion per formula unit on charge, corresponding to about 165 mAh/g specific capacity at C/16 rate. After a few cycles, capacity around 140 mAh/g was maintained. However, some impurity phases also co-exist with Li_2_FeSiO_4_, which cause side reactions during cycling.

**Figure 3:**
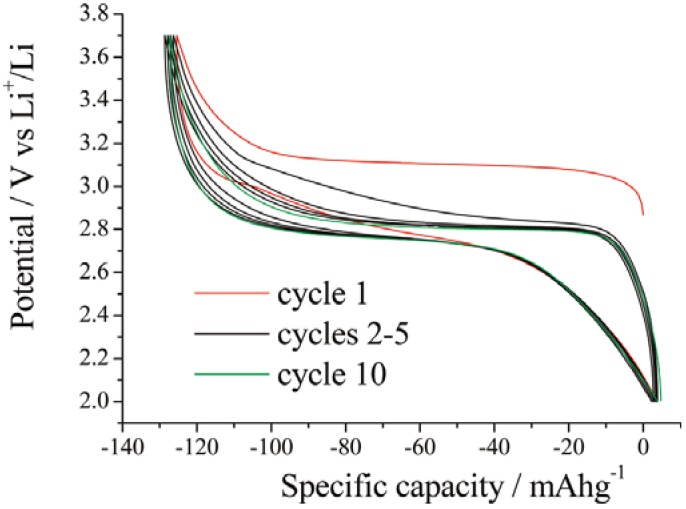
Variation of potential with state of charge on cycling Li_2_FeSiO_4_ at a rate of 10 mA/g (C/16) and demonstrating the change of potential and polarization upon cycling. Adapted with permission from [29]. Copyright 2011 American Chemical Society.

Initial charge profiles of Li_2_FeSiO_4_ were above 3 V, and subsequent charge curves shifted to lower voltage around 2.9 V, as shown in Figure 3. Structural rearrangements involving the site exchange of lithium and iron during the first charge was confirmed by X-ray diffraction technique [28,29].

Quite interestingly, Li/Fe site-mixing does not lead to any capacity loss or rate performance deterioration. The density functional theory (DFT) approach suggests such mixing does not appear to impede the Li vacancy migration; it may even open alternative pathways for effective Li-migration in all three dimensions [30]. The intrinsically poor conductivity of LiMPO_4_ (M = Fe, Mn, Co) family materials has been overcome by decreasing particle size and coating them with conductive carbon. A similar strategy was also applied to orthosilicates Li_2_MSiO_4_. nano-particles and carbon coating are both beneficial to the performance of Li_2_MSiO_4_. The as-prepared Li_2_MnSiO_4_ material was usually electrochemically inactive unless coated with a significant amount of carbon. Belharouak *et al.* synthesized carbon-coated and ball-milled nanosized (100 nm) Li_2_MnSiO_4_ by the sol-gel method [31]. Capacities of 190 and 172 mAh/g were achieved, respectively; however, only less than 100 mAh/g discharge capacities were retained after 15 cycles. Gong *et al.* synthesized uniform 40–80 nm Li_2_FeSiO_4_ nanoparticles by the hydrothermal-assisted sol-gel process [32]. Carbon-coated Li_2_FeSiO_4_ as an electrode material displays a large discharge capacity of ca. 160 mAh/g at C/16 rate. The electrodes are also capable of delivering a specific capacity of 125 mAh/g at a high current of 2C rate.

**Figure 4:**
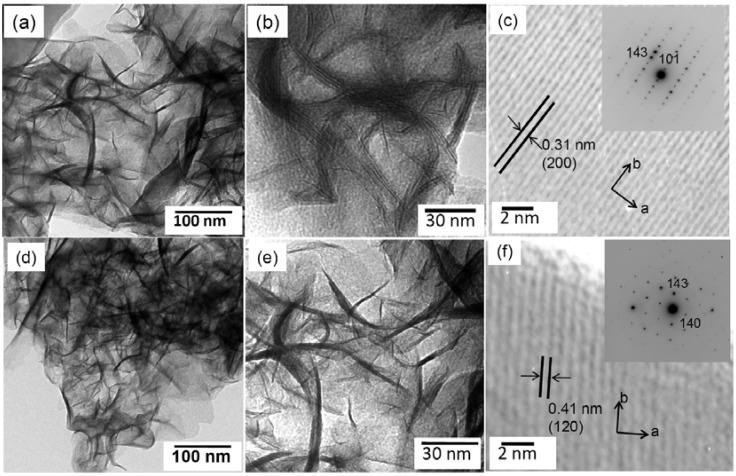
Transmission electron microscopy (TEM) images of the as-prepared samples. (**a**,**b**) L_i2_FeSiO_4_; (**d**,**e**) Li_2_MnSiO_4_ nanosheets; (**c**,**f**) High-resolution transmission electron microscopy images (HRTEM) of Li_2_FeSiO_4_ and Li_2_MnSiO_4_ nanosheets with selected area electron diffraction (SAED) patterns in the inset, respectively. Adapted with permission from [35]. Copyright 2012 American Chemical Society.

**Figure 5:**
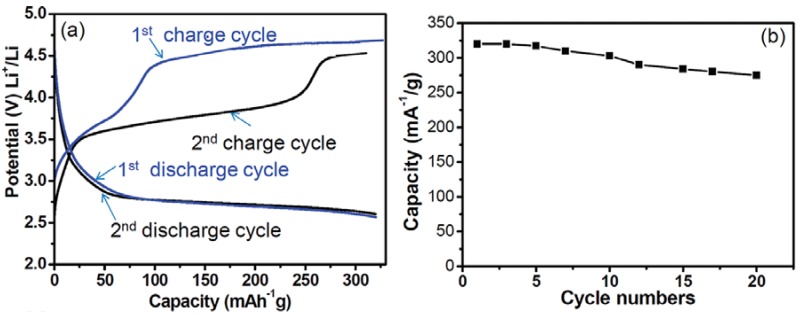

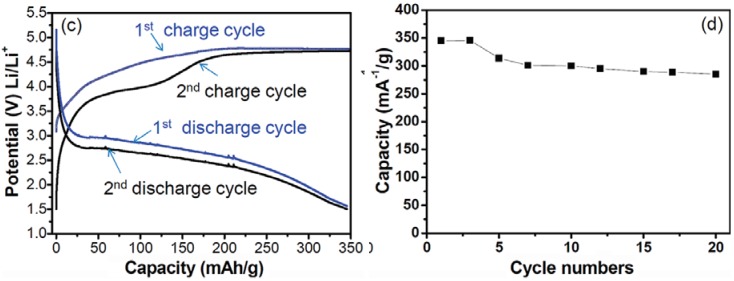
Charge and discharge profile of first and second cycles. (**a**,**c**) Li_2_FeSiO_4_ and Li_2_MnSiO_4_ at 45 °C, cycling at a current rate of C/50; (**b**,**d**) The cycling performance of Li_2_FeSiO_4_ and Li_2_MnSiO_4_, respectively. Adapted with permission from [35]. Copyright 2012 American Chemical Society.

Although capacities higher than one Li-ion per formula unit (170 mAh/g) are desirable, Li_2_MnSiO_4_ typically only exhibits a reversible insertion/extraction of less than 0.5 lithium ion per formula unit (85 mAh/g). Amorphization of Li_2_MnSiO_4_ during cycling was proposed as the reason of drastic capacity fading for the subsequent electrochemical cycling. DFT calculations show Li_2_MnSiO_4_ structure may collapse upon removal of large amounts of lithium. Jahn–Teller distortion associated with the Mn^3+^ may also contribute to the instability of the structure [33]. Aravindan *et al.* have recently employed carbon-Li_2_MnSiO_4_ composite cathodes to improve electrochemical performance [34]. They found the electrochemical performances of these composites are greatly affected by the carbon content. Composite electrode, which has a 42% carbon content, exhibited a very stable discharge behavior 140 mA h/g for 40 cycles at room temperature. Reducing carbon content to less than 30% will lead to much lower capacity (75 mAh/g) at the same condition. However, such a high amount of carbon adversely affects the tap densities of Li_2_MnSiO_4_ and further decreases volumetric energy density. Very recently, Rangappa *et al.* reported that Li_2_FeSiO_4_ and Li_2_MnSiO_4_ electrodes with 2D nanostructures (nanosheets) exhibit a promising two-lithium extraction/insertion performance at elevated temperature (45 ± 5 °C) with a good cycle ability up to 15−20 cycles [35]. Li_2_FeSiO_4_ and Li_2_MnSiO_4_ nanosheets were successfully synthesized by one-pot supercritical fluid reaction using a mixed solvent of aqueous ethanol at 400 °C for 10 min. The morphology of these nanosheets were observed by transmission electron microscopy (TEM), as displayed in Figure 4. As-prepared Li_2_MSiO_4_ are almost electrochemically inactive. In order to increase the conductivity, as-synthesized materials were ball-milled with conductive polymer poly(3,4-ethylenedioxythiophene) and multiwall carbon nanotubes (MWCNT), followed by mild heat treatment at 300 °C for 3 h under Ar atmosphere. A discharge capacity of 160 mAh/g for the Li_2_FeSiO_4_ and 220 mAh/g for the Li_2_MnSiO_4_ electrode was achieved at room temperature. Although large polarization is observed, 340 and 350 mAh/g (corresponding to two lithium ions per formula unit) at 45 ± 5 °C has been achieved at the very low rate of C/50. Figure 5 shows the electrochemical performance of Li_2_FeSiO_4_ and Li_2_MnSiO_4_ at 45 ± 5 °C. Drastic capacity fading was observed after 20 cycles to retain only 40%−50% of the initial capacity. However, the result is encouraging, as it shows the possibility of achieving high capacity in the Li_2_MSiO_4_ system.

Although Li_2_C_O_SiO_4_ can be a potential high voltage cathode, there has been less interest, because of the toxicity and cost of cobalt. A theoretical study suggested that an extraction of second lithium from Li_2_C_O_SiO_4_ would occur at a high voltage of around 5 V [36]. So far, no successful results have been reported due to the lack of electrolytes in lithium batteries with high stability (up to 5 V). Gong *et al.* prepared Li_2_CoSiO_4_ by solution and hydrothermal synthesis [37]. Li_2_CoSiO_4_/C composite materials show a large irreversible capacity during the initial cycle. One-point-four Li^+^ per unit formula (corresponding to a capacity of 234 mAh/g) was able to be extracted. However, only less than half of the lithium can be inserted back. Thus, a charge capacity beyond one lithium per unit formula may come from electrolyte decomposition. Lyness *et al.* studied three polymorphs of Li_2_CoSiO_4_ [38]. After carbon coating and cycling these materials between 2.0 and 4.6 V at 10 mA/g, some reversible capacity was obtained. Similar to Gong’s report, over 64% irreversible capacity losses of these materials were observed during the first cycle. The capacities also faded quickly and only maintained less than 60 mAh/g after 10 cycles.

Silicate materials, especially Li_2_FeSiO_4,_ have shown some promising properties as candidates for next generation Li-ion cathode materials. Now, it is possible to achieve a two lithium ions insertion and extraction at 50 °C. The remaining challenge is how to improve the electrochemical performance (with a multiple-electron exchange process) of these materials at room temperature.

## 3. Anodes

Graphite, a layered intercalation compound, is one of the most widely used lithium anodes. Lithium can be readily inserted and extracted into the structure. The lithiated compounds have stable phases up to the LiC_6_ stoichiometry corresponding to a theoretical specific capacity of 372 mAh/g. Tin and silicon based materials offer specific capacity values much higher than conventional graphite. Unfortunately, all of these classes of materials are generally plagued by large volume expansion during lithiation, which generates enormous mechanical stress and pulverizes the electrode during the charge/discharge cycles. Besides the intercalation compounds and metal alloys (such as tin and silicon based materials), another category of anodes are based on a conversion reaction, such as transition-metal oxides (MO, where M is Co, Ni, Cu or Fe). They have been intensively studied and reviewed elsewhere [39,40,41,42]. In spite of a high specific capacity, conversion compounds usually suffer from large voltage hysteresis between charge and discharge, which severely retards the round-trip efficiency of the electrode. Recent advancements of nanostructured materials afford new opportunities to improve the current technology for improving both the rate capability and cycliablity of Li-ion anodes. Over the past five years, significant progress has been made for the development of these lithium metal alloy anodes with high energy density. In this section, these efforts to improve the electrochemical performance are reviewed.

### 3.1. Tin-Based Materials as Advanced Anode Materials for Lithium Ion Batteries

#### 3.1.1. Pure Tin Anode

The theoretical capacity of pure Tin is 994 mAh/g, which is three times that of the graphite anode (372 mAh/g), based on the end lithiated phase Li_4.4_Sn. Later, crystallographic studies suggested that the realistic form of this end phase could be Li_17_Sn_4_ (thus, 4.25 Li per Sn [43,44]). Therefore, its maximum gravimetric capacity could be 959.5 mAh/g, which is still much higher than most common graphite anodes. Also, the potential of the tin-based anodes is slightly higher than that of graphite, which reduces the potential safety problems with metallic lithium deposition on the host anode, which occurs during rapid charging and discharging. Unfortunately, they are plagued by the large volume change of these metals during lithium insertion/extraction. Decrepitation or fracture of particles in an electrode into smaller pieces normally happens during the intercalation/de-intercalation of lithium ions. About a 360% volume expansion of pure tin causes the internal strain of the materials.

Demonstrated by the Whittingham group, pure tin foil (bulk) can be cycled as 600 mAh/g for 10 to 15 deep cycles [45]. However, the expansion and contraction of the electrode crystalline lattice cause some breaking-up of the material and are responsible for the increase of the cell impedance. Consequently, the loss of electronic contact between active materials and the current collector dramatically decreases the reversible capacity after 15 cycles. In order to overcome the decrepitation effects, nanostructures or nanocomposite have been extensively investigated. Theoretically, the volume change during electrochemical cycling may be under control, and the lithium diffusion length can be greatly reduced when the tin particle size is at a nanometric level. The absence of dislocations in nanometric materials makes them less susceptible to cracking and decrepitation. In addition, dispersion of the tin particles in a matrix-forming nanocomposite is also very effective to buffer the large volume change. Tin-based anodes have demonstrated enhanced performance and improved cycle life with nanoscale or composite particles.

#### 3.1.2. Tin/Carbon Composite

Earlier studies of using physical mixing of carbon and tin oxide or simply mixed tin precursor salts with carbon precursor does not result in homogenous distribution of tin in the composite [46,47]. Kim *et al.* reported Sn/C composite by infiltrating tetraethyltin into mechanically milled polystyrene resin powder [48]. The resultant composite exhibited a capacity of 480 mAh/g with good capacity retention over 30 cycles. Recently, Hassoun *et al.* were able to synthesize carbon-tin composites by infiltration of a tin precursor into an organic gel, followed by calcination under argon [49]. Uniform dispersed tin particles were found in the carbon matrix. The particle size of tin was around 50nm at the surface and less than 10 nm in the bulk. Also, a small amount of residual SnO_2_ (8.8%) coexisted in the composite. Smartly designed nano-composites demonstrated excellent cycling performance. At a 0.8 C rate, the electrode delivers a specific capacity on the order of 500 mAh/g and remains stable over more than 200 cycles. At 5 C rate, the composite recovered 200 mAh/g, which is about 40% of its total capacity. Later, Scrosati *et al.* further improved the synthesis procedures and obtained a pure tin compound without a trace of tin oxide. The tin component in the composite is highly stable stored in open air without undergoing any decomposition, because of the protective carbon matrix. The high purity Sn–C composite delivers a reversible and stable capacity on the order of 450 mAh/g. However, all of these composites have 50% carbon content, which reduced the composite volumetric energy capacity by half, and an additional 10% Super P carbon during electrode preparation lowered its density further.

Very recently, Luo *et al.* reported that tin-core/carbon-sheath coaxial nanocables were fabricated by the CVD process using reduced graphene oxide (RGO)-based hybrid material as an efficient platform [50]. The tin content in the nanocable samples was determined to be 61.0 wt %. Cycling at a current density of 100 mA/g, the RGO-Sn–C nanocables had specific capacities higher than 760 mAh/g in the initial 10 cycles and higher than 630 mAh/g after the 50th cycle.

#### 3.1.3. Tin–(M)–Carbon (M = Co, Fe, Ti)

SONY’s recent commercial success of the Nexelion battery, which was based on a novel nanostructured anode, was shown to contain nanosized amorphous SnCo embedded in carbon [51]. Sn–Co exists as 5-nm particles encapsulated in carbon, which most likely prevents much contact between the electrolyte and the metal. Thus, few side reactions happened during electrochemical cycling. Also, the electrochemical behavior of the amorphous tin anode was found to be superior to that of crystalline tin-based materials [52]. Heating the sample in H_2_/He atmosphere can crystallize the SnCo amorphous material and also maintain carbon content. The crystallization primarily happened at 300 °C. When the amorphous Sn-Co carbon composite were heated to 250 °C, the capacity of the material was about 300mAh/g for the initial cycle and rapidly dropped to about 200 mAh/g after 10 cycles. After heating to 450 °C, the capacity is only about 50 mAh/g. Despite its remarkable performance, cobalt is expensive and toxic.

Recently, the Whittingham group reported Sn/Fe/C composite prepared by mechanical milling using Ti, Al and Mg as the reducing agent and different grinding media [52]. The specific capacity of 600 mAh/g, close to the theoretical capacity, was obtained on titanium reduced Sn-Fe carbon composite with a good capacity retention on cycling 200 cycles. Also, Sn-Fe-carbon composite has a comparable rate capability with Sn-Co-C materials at a current density of 5 mA/cm^2^. It still can achieve a specific capacity of 250 mAh/g when charged and discharged between the 0.01–1.5V voltage window.

#### 3.1.4. Tin Oxide Materials

Tin oxide materials were first introduced as promising anode materials by the Fuji amorphous tin-based composite oxide [53]. The tin-based composite oxide (TCO) has a basic formula SnM*_x_*O*_y_* (M = B, P, Al). About eight equivalent moles of Li-ions per unit mole can be alloyed with the TCO during discharge (Li insertion), which corresponds to a specific capacity of 1030 mAh/g. After initial charge (Li extraction), the reversible capacity of 650 mAh/g can be achieved with a coulombic efficiency close to 100%. The working potential of TCO (between 0 to 1.2 V *versus* Li/Li^+^) makes it very suitable for use as an anode material. Since Idato *et al.* reported on TCO, tin oxide materials have drawn extensive attention because of their large capacity, environmental acceptability and low cost. The reaction mechanism of SnO_2_ with lithium has been proposed as the following two steps:

SnO_2_ + 4Li^+^ + 4e^−^ => Sn + 2Li_2_O (1)

Sn + *x*Li^+^ + *x*e^−^ ↔ Li*_x_*Sn (0 ≤ *x* ≤ 4.4) (2)

In the first process, SnO_2_ was reduced to metallic Sn, which may be partially irreversible. The second process is the Sn alloying/de-alloying reaction with lithium, which is very reversible in most cases. If completely reversible, the overall electrochemical processes could lead to a total of 8.4 Li-ions insertion per formula unit of SnO_2_. This corresponds to a theoretical capacity of 1491 mAh/g. If only assuming the second step is reversible, as many as 4.4 Li atoms can be stored per Sn atom, which still gives the theoretical capacity of 781 mAh/g.

Besides the problem of SnO_2_ particle pulverization during cycling, agglomeration of the primary particle will drastically reduce the surface-to-volume ratio and, thus, diminish the electrochemical activity of tin oxides. Several strategies have been utilized to mitigate the pulverization and particle agglomeration, e.g., nanomaterials with a hollow structure for providing the “breadth” space. In the past decade, great efforts have been devoted to synthesizing SnO_2_ nanostructures with different morphologies.

##### 3.1.4.1. Tin Oxide Nanoparticles

Kim *et al.* reported that nanocrystalline SnO_2_ in various crystallite sizes (3 nm, 4 nm and 8 nm) was synthesized via a hydrothermal reaction under various temperatures [54]. Particles with a size of about 3 nm showed excellent performance over 60 cycles, with an initial charge capacity of 740 mAh/g. The size effect was obvious; the 3 nm SnO_2_ outperformed the 4nm and 8nm particles both in specific capacity and capacity retention. Negative electrodes of 4 and 8 nm-sized showed an initial capacity of 636 and 554 mAh/g, and their capacity retentions were only 73% and 3% after 60 cycles, respectively. High-resolution transmission electron microscopy (HRTEM) analysis revealed that SnO_2_ nanoparticles with about 3 nm experienced a reversible volume change without aggregation into larger Sn clusters during cycling. However, larger nanoparticles tend to aggregate after several cycles. Finally, significant (more than 60%) first cycle losses were observed for all these nanoparticles due to a side reaction between electrode and electrolyte.

Recently, Want *et al.* prepared Sn/SnO*_x_* core-shell nanospheres by polyol synthesis [55]. By controlling the polymer surface stabilizer (PVP) and reaction temperature, the nanosized-Sn/SnO*x* core-shell (30, 45 and 79 nm) and the sub-micro-sized-Sn/SnO*_x_* (100–500 nm) were obtained. After cycling, micro-sized (0.3–10-*μ*m or 100–500 nm) particles have severe problems, *i.e.*, coarsen, pulverization and adhesion loss to the conductive carbon matrix. Compared with 0.3–10-*μ*m particles, no obvious decrepitation of 45 nm Sn/SnO*_x_* core-shell nanospheres were observed. However, aggregation does occur in the 45nm particle. With the cycling rate of C/20 based on the theoretical capacity of tin, 45 nm Sn/SnO*_x_* core-shell nanospheres exhibit a good specific capacity of about 550 mAh/g. The volumetric and gravimetric capacity after 10 cycles was about 3443 mAh/cm^3^ and 480 mAh/g, respectively. These are about 88% of the initial capacity. Unfortunately, the capacity faded quickly after 10 cycles and remained quite low. These results also suggest that the critical size of SnO_2_ to prevent both pulverization and aggregation should be less than 10 nm.

##### 3.1.4.2. Tin Oxide Hollow Structures

SnO_2_ hollow structures have been extensively studied for application in Li-ion battery. The additional void space in the hollow interior can be useful to accommodate a large strain from the materials during electrochemical cycling. Lou *et al.* reported a one-pot synthesis of SnO_2_ hollow nanospheres [56]. The synthesis was performed in a mixed solvent of ethanol and water using potassium stannate (K_2_SnO_3_) as the precursor and urea as an additive. The hollow structured SnO_2_ wasformed during the Inside-out Ostwald ripening process. The BET surface area of these SnO_2_ hollow nanospheres is 110 m^2^/g. SnO_2_ nanospheres demonstrated excellent electrochemical performance. At a rate of 0.2 C, when charged to 2 V, a specific capacity of 1140 mAh/g was observed, which is higher than the theoretical value based on tin alloyed and de-alloyed with lithium. Lou *et al.* attribute the performance to the highly porous shell and the hollow interior, which may facilitate the surface storage of extra lithium. Although capacity faded upon cycling, half of the capacity was maintained over 30 cycles.

Deng *et al.* reported their synthesis of SnO_2_ nanoparticle aggregates with a mesoscale hollow core-shell structure [57]. By using glucose and SnCl_4_ in a water/ethanol mixture as the starting materials, the solvothermal reactions were carried out in the autoclave at 180 °C for 24 h. The hollow core–shell structured nanoparticles exhibited good electrochemical performance and achieved high first cycle charge (lithiation) and discharge (de-lithiation) capacities of about 2300 mAh/g and 1300 mAh/g, respectively. Similar to Lou’s report, the materials show reduced specific capacity after several cycles.

**Figure 6:**
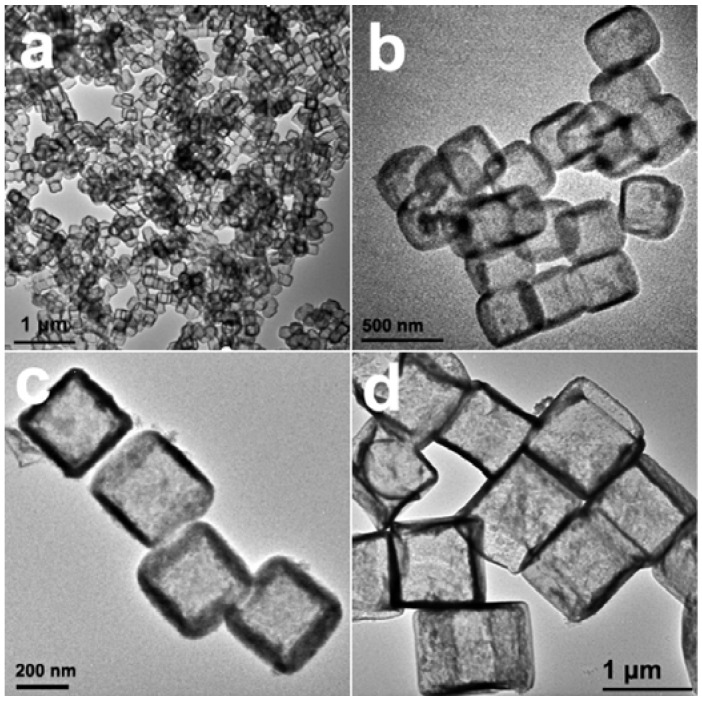
TEM images of SnO_2_ nanoboxes with different edge lengths: (**a**–**c**) 200–250 nm; (**d**) 800–1000 nm. Adapted with permission from [58]. Copyright 2012 American Chemical Society.

**Figure 7:**
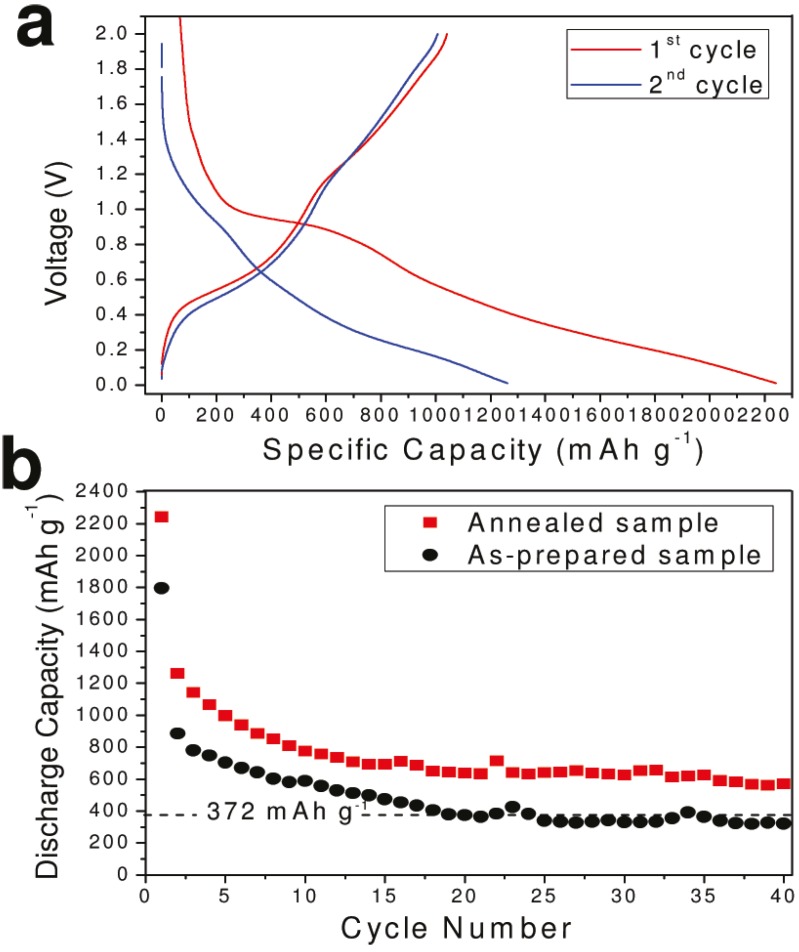
(**a**) Discharge/charge voltage profiles of annealed SnO_2_ nanoboxes cycled between 0.01 and 2.0 V at a 0.2 C rate; (**b**) cycling performance of as-synthesized and annealed SnO_2_ nanoboxes at 0.2 C. Adapted with permission from [58]. Copyright 2012 American Chemical Society.

Non-spherical SnO_2_ hollow structures are not commonly reported, due to the lack of non-spherical templates. Recently, uniform SnO_2_ nanoboxes were synthesized by the etching of Cu_2_O nanocubes as sacrificial templates (Figure 6) [58]. Simultaneously controlled hydrolysis of Sn^4+^ and coordinated etching of pre-grown Cu_2_O nanocubes results in 200 to 800 nm particles. Electrochemical testing of these annealed SnO_2_ nanoboxes at a 0.2 C rate was within a cutoff window of 0.01–2.0 V. As shown in Figure 7, the nano-boxes exhibited a high initial capacity of 1041 mAh/g. After 40 cycles, a 45% capacity loss was observed and about a specific capacity of 570 mAh/g remained. The as-synthesized nanoboxes (without annealing) have a much faster capacity decay, as the capacity drops to around 370 mAh/g even after 20 cycles.

##### 3.1.4.3. Tin Oxide Nanotubes

**Figure 8:**
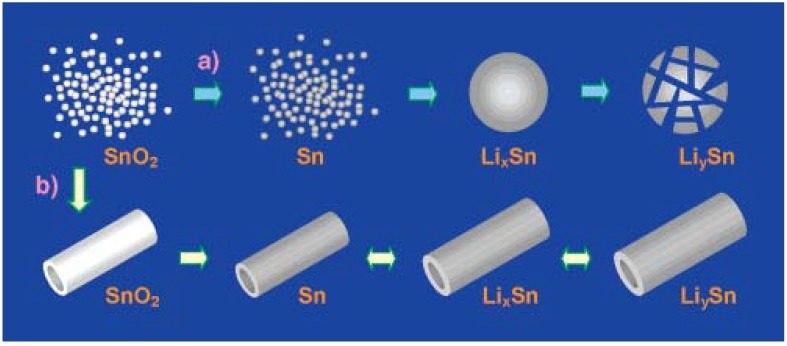
Schematic illustrations of two different fabrications of SnO_2_: (**a**) SnO_2_ nanoparticles without preorganization cause aggregations and pulverization of Li*_y_*Sn*_x_* (assuming *y,x*) upon cycling; (**b**) when nanocrystallites of SnO_2_ are organized into a tubular configuration, concentric expansion and contraction could be expected upon lithium charging and discharging. Adapted with permission from [59]. Copyright 2005 American Chemical Society.

Nanotube morphology also has advantages to improve the performance of SnO_2_. First, cavities of the nanotubes usually can provide a sufficient space to sustain the volume change. Second, porous external and internal surfaces promote the Li-ion transportation. Last, but not least, the one dimensional tubular structure in the nanotube confines the movement of SnO_2_ and much less aggregation could occur, as shown in Figure 8. Anodic aluminum oxide (AAO) and the polycarbonate (PC) membrane template method are commonly used as sacrificial templates for making nanotubes. Wang *et al.* developed a method to make SnO_2_ nanotube by the template method [59]. Commercially available SnO_2_ nanoparticles were infiltrated into the membrane channels and calcined to a dense packaging of SnO_2_ nanoparticles on the walls of the AAO membrane. Subsequently, the AAO template was removed. At a low current density of 0.05 mA/cm^2^ with a cutoff voltage window of 5 mV to 2 V, uniform polycrystalline SnO_2_ nanotubes exhibited a high initial capacity of 940 mAh/g. After 80 cycles, SnO_2_ nanotubes retain 55% of the initial capacity, which was 525 mAh/g.

Ye *et al.* successfully prepared SnO_2_ nanotubes by using one-dimensional (1D) silica mesostructures as sacrificial templates [60]. Cycling this material at a current of 100 mA/g with a cutoff voltage window of 5 mV to 1.5 V, the specific capacity of 468 mAh/g was retained after 30 cycles. The average capacity fading of these SnO_2_ nanotubes is about 1.7% to 2.1% after the second cycle, which is much smaller than that of SnO_2_ nanopowders. The improved performance might be related to the robust hollow structure of short nanotubes that is favorable for alleviating volume changes and mechanical stress during charging/discharging processes.

##### 3.1.4.4. Tin Oxide Nanowires

Liu *et al.* synthesized SnO_2_ nanowires by the self-catalysis growth method [61]. Obtained SnO_2_ nanowires are randomly aligned with diameters of 200–500 nm. The SnO_2_ nanowires show improved performance compared to SnO_2_ powder. Liu *et al.* contribute the enhanced performance to more reaction sites on the surface in the nanowires. Park *et al.* further investigated morphology effects on the electrochemical performance of nano-sized SnO_2_ materials [62]. Over the voltage range of 0.05 V to 1.50 V, both nanowires and nanotubes outperformed the nanoparticles at a current density of 100 mA/g. Although all showed coulombic efficiencies less than 50%, both SnO_2_ nanowires and nanotubes show initial coulombic efficiencies of about 47% and 40%, respectively. SnO_2_ nanopowders have an average particle size of 100 nm and show only about 31% coulombic efficiency during the first cycle. The SnO_2_ nanowire and nanotube electrodes exhibited a higher reversible specific capacity than 100 nm SnO_2_ nanopowders, up to the 15th cycle (300 mAh/g and 100 mAh/g, respectively). The authors explained that the enhanced rate capability and cycling performance of the electrode could be attributed to the prevention of undesirable Li-ion trapping or loss of electronic connection between active materials caused by volume variation.

##### 3.1.4.5. Tin Oxide Nanosheets

The two-dimensional (2D) anisotropic structure of SnO_2_ may have the benefit of structural flexibility against the volume variation during the charge-discharge process. Wu *et al.* reported nanosheets of SnO_2_ synthesized by a solvothermal method in a mixed solvent of water and ethanol. Three-dimensional (3D) hierarchical structures assembled from nanosheets were obtained. At a relatively high current rate of 800 mA/g, a specific capacity of 400 mAh/g can be retained for 30 cycles. It was assumed that the short lithium diffusion path was responsible for the improved electrochemistry [63]. Chen *et al.* also prepared micro-sized flower-like structures assembled from large highly crystalline phase-pure SnO_2_ nanosheets [64]. They found that the presence of ammonium fluoride (NH_4_F) played a very important role for the formation of the nanosheets structure during the hydrothermal treatment of SnCl_2_ aqueous solution. Although high initial irreversible capacity loss (over 60%) was observed, the material showed good electrochemical performance. Cycling between 0.01–1.2 V, the specific capacity of nanosheets was maintained above 450 mAh/g for over 50 cycles at current density of 400 mA/g.

##### 3.1.4.6. Tin Oxide/Carbon Composite

The beneficial effect of carbon addition as a matrix or coating has been widely observed in both Li-ion cathode and anode materials. Significant work has been done for improvement of LiFePO_4_ cathode materials by carbon addition or coating. Now, LiFePO_4_/C composite materials have been successfully commercialized. For the tin anode system, carbon can act as the conductor and as the structural buffer. Carbon additives or coating can provide good electric conductivity for the SnO_2_. It also buffers against particle volume expansion during lithiation and, thus, limited the pulverization of the particles. Enhanced mechanical stress tolerances of the composite help the SnO_2_ achieve larger capacity and a better cycle life. Various approaches have been explored to produce SnO_2_/C composite. Some approaches are very simple. For example, SnO_2_–carbon composites can be formed similarly to coating on the LiFePO_4_ using the hydrothermal coating method with glucose as the carbon source [13,65]. Lee *et al.* synthesized tin-encapsulated spherical hollow carbon by the pyrolysis method, which showed enhanced electrochemical performance [66]. However, because of the relatively low content of Sn (only about 24 wt %) and thick carbon shell, the specific capacity was limited to about 400 mAh/g. Zhang *et al.* designed an approach for forming tin-nanoparticles encapsulated in elastic hollow carbon spheres (TNHCs) [67]. The synthetic procedure involves forming SnO_2_ hollow spheres from sacrificial template SiO_2_ and *in situ* carbon coating by hydrothermal treatment. The tin oxide carbon nanocomposites have a specific capacity of >800 mAh/g in the initial 10 cycles and >550 mAh/g after the 100th cycle. Due to both the 70%–80% void volume of the carbon shell and the elasticity of the thin carbon spherical shell (about 20 nm), it can efficiently accommodate the volume change of tin nanoparticles due to the Li-Sn alloying-dealloying. To simplify the carbon coating process of SnO_2_/C composite, Lou *et al.* performed one-pot synthesis of SnO_2_–C nanocolloids by the hydrothermal method [68]. The starting material K_2_SnO_3_ and glucose were hydrothermally treated. Glucose not only serves as the carbon source, but also facilitates precipitation of SnO_2_. SnO_2_/C nanocolloids showed improved capacity retention compared to the pristine SnO_2_. At a current density of 300 mA/g, a capacity of ca. 440 mA h/g can be obtained after more than 100 cycles.

Graphene, a monolayer of graphite, has a very high specific surface area (theoretical value 2620 m^2^/g), electronic conductivity and good elasticity. Graphene sheets distributed between the SnO_2_ nanoparticles can prevent their direct contact and thereby minimize the aggregation and pulverization of the SnO_2_ nanoparticles. Thus, enhanced lithium storage properties compared to the pure SnO_2_ nanoparticles are usually observed. Wang *et al.* prepared SnO_2_-graphene nanocomposite by ternary self-assembly approaches [69]. The nanocomposite (60 wt % SnO_2_ and 40 wt % graphene) showed stable electrochemical cyclability. At a low current of 10 mA/g, a reversible capacity of 520 mA h/g was maintained over 100 cycles between 0.02 and 1.5 V. Zhang *et al.* reported that monodispersed SnO_2_ nanoparticles can be deposited on both sides of single-layer graphene sheets [70]. Benefitting from the synergetic effects of ultrafine SnO_2_ nanoparticles and the conductive graphene nanosheets, the composite exhibited a high reversible capacity of 786 mAh/g and a 71% retention of its initial capacity in the range of 0.02 to 3 V (*vs.* Li/Li^+^) after 50 cycles. Ding *et al.* directly grew SnO_2_ nanosheets on a graphene oxide support and subsequently reduced graphene oxide to graphene [71]. The electrochemical properties of Graphene-SnO_2_ nanosheets as an anode were performed at a relatively high current rate of 400 mA/g. Although relatively high irreversible capacity (>60%) is still observed in the first discharged capacity, Graphene supported–SnO_2_ material manifests a much enhanced cycling performance compared to pure SnO_2_ nanosheets. The specific capacity of 518 mAh/g can still be deliverable after 50 cycles.

Carbon nanotubes also have been suggested as the choice of carbon to improve the SnO_2_ electrochemical performance, because of the one-dimensional (1D) tubular structure and high electrical conductivity. Wang *et al.* fabricated SnO_2_ nanotubes first and subsequently grew a CNT overlayer on the external surface of the SnO_2_ nanotubes through a confined-space catalytic deposition process [72]. In the wide voltage window of 5 mV–3 V, the SnO_2_–CNT nanocomposite exhibited highly reversible capacity (540–600 mAh/g), with a capacity retention of 92.5% after 200 charge and discharge cycles. The tubular organization of SnO_2_ nanoparticles with the carbon nanotube limits the mobility of particles. The large internal void in the core-shell construction buffers well against the volume expansion. These unique features of this nanocomposite might be responsible for excellent electrochemical cyclability. Wen *et al.* also synthesized mesoporous SnO_2_ with a cassiterite structure through the hydrothermal method using cetyltrimethylammonium bromide (CTAB) as structure-directing agents [73]. The hybrid structure was formed by *in situ* growth of mesoporous SnO_2_ on the surface of multi-walled carbon nanotubes (MWCNTs). At a relatively low current density of 33.3 mA/g, MWCNT hybrid electrodes delivered an initial discharged capacity of 622.9 mAh/g. The capacity dropped to 460 mAh/g at the second cycle, and SnO_2_/C composite showed around a 350 mAh/g capacity maintained over 50 cycles. However, the mesoporous SnO_2_ remained only at 160 mAh/g after 50 cycles.

##### 3.1.4.7. *In situ* Observation of the Electrochemical Lithiation of Tin Oxide Electrode

So far, fundamental physical and chemical changes, which take place during SnO_2_ lithiation/de-lithiation are not well understood. Most of the structure or microscopic studies are *ex situ* experiments and provide little information for understanding the mechanism. Observation of real-time processes of lithiation is crucially important for the future developments of these electrode materials. Recently, Huang *et al.* demonstrated that *in situ* TEM (transmission electron microscopy) is capable of observing the real-time lithiation process of a single SnO_2_ nanowire [74]. The simplified electrochemical cell comprised a single SnO_2_ nanowire as the anode, ionic liquid as the electrolyte and bulk lithium cobalt oxide as the cathode. The ionic liquid LiTFSI, which has a very low vapor pressure, enabled its use as an electrolyte under high vacuum for *in situ* TEM study. During discharging (lithiating) of the cell, the lithiation reaction front propagated progressively along the nanowire. To accommodate the large volume expansion, the nanowire swelled, elongated and spiraled. Huang *et al.* observed elongation of 90%, a diameter expansion of 35% and a total volume expansion of 250% in SnO_2_ nanowire by TEM. SnO_2_ was also found to be first reduced to nanocrystalline Sn and amorphous Li_2_O. Similar to bulk SnO_2_ anode, the formed Sn then reacts with lithium as follows: Sn + *x*Li^+^ + *x*e^−^ ↔ Li*_x_*Sn (0 ≤ *x* ≤ 4.4). The demonstrated single SnO_2_ nanowire seems to be able to accommodate the volume expansion quite well by elongation and spiration without fracturing. The amount of deformation occurring during discharging and charging might wear down SnO_2_ nanowires after a certain number of cycles. Even so, the SnO_2_ appeared to be far better as a nanowire than in its larger, bulk form.

#### 3.1.5. Tin-Based Intermetallic Anode Materials

**Figure 9:**
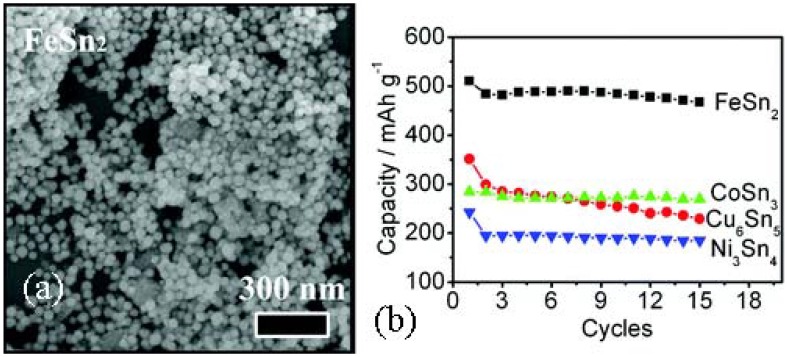
(**a**) Scanning electron microscopy (SEM) image of FeSn_2_; (**b**) Cycling performances of intermetallic nanospheres at C/20 rate adapted with permission from [75]. Copyright 2012 American Chemical Society.

Besides forming tin/carbon composite, active host matrix can effectively improve the electrochemical cycling of the Sn anode materials. In the Sn-based intermetallic compound, the second metal (M) may buffer the volume change by forming a soft framework, which stabilizes the integration of a single intermetallic particle and enhances the electronic conductivity during cycling [75]. A uniform particle size of ≈40 nm single-crystalline Sn-based intermetallic compound (M = Fe, Cu, Co, Ni) nanospheres have been synthesized via the polyol process, as displayed in Figure 9a. FeSn_2_, Cu_6_Sn_5_, CoSn_3_ and Ni_3_Sn_4_, all show good electrochemical performance. Surprisingly, the volume discrepancy between the charged (delithiated) and discharged (lithiated) phases seems to have little influence on the cycling performance. FeSn_2_ nanospheres show a top capacity of about 500 mAh/g, maintained for at least 15 cycles, as shown in Figure 9b. Assuming the lithiated product of those Sn-based intermetallic compounds to be L_i4.4_Sn, the volume of Li_4.4_Sn is 340% that of FeSn_2_ as compared to 238% for Cu_6_Sn_5_, 308% for Ni_3_Sn_4_ and 349% for CoSn_3_. However, the capacities of these intermetallic nano-spherical compounds investigated can be listed in the following order: FeSn_2_ > Cu_6_Sn_5_ ≈ CoSn_3_ > Ni_3_Sn_4_. The better electrochemical performance of FeSn_2_ anode may stem from its crystal structure. The open channels located within the FeSn_2_ crystal lattice promote the penetration and alloying of Li^+^ within the Sn host.

A new FeSn_5_ phase was recently reported. The material crystallized in a tetragonal phase with a P_4_/mcc space group [76]. The new phase is nonstoichiometric with a large number of Fe vacancies present, and the formula could be written as Fe_0.74_Sn_5._ A Sn/Fe molar ratio of 6.8 was determined by Rietveld refinement and energy dispersive X-ray spectroscopy (STEM-EDS). The presence of Li storage-inactive Fe in FeSn_2_ lowers its theoretical capacity to about 800 mAh/g. Compared to FeSn_2_, Fe_0.74_Sn_5_ has a significantly lower Fe to Sn ratio and has a theoretical capacity of 929 mAh/g, which is the highest to date for the reported M and very close to the theoretical capacity of pure tin. Fe_0.74_Sn_5_ nanospheres showed excellent performance as an anode in Li-ion batteries. At a low current density (C/20), it can maintain a capacity of 750 mAh/g for 15 cycles, which is higher than the 500 mAh/g of common FeSn_2._

### 3.2. Silicon-Based Materials as Advanced Anode Materials for Lithium Ion Batteries

#### 3.2.1. Pure Silicon Anode

Silicon possesses a very high theoretical capacity of 4200 mAh/g. Unfortunately, similar to tin anode, silicon also suffers from huge volume expansion upon cycling. A volume expansion of 400% occurs upon insertion and extraction of lithium during cycling [77,78,79]. This is almost one of the highest volume expansions among the common alloy anodes. Thus, very high irreversible capacity in the first cycle and capacity fading of subsequent cycles were observed for the micron size silicon. Ryu *et al.* showed that commercial bulk silicon powder consists of 10 um particles as anode materials, and the first discharge capacity (lithiation) was about 3260 mAh/g [80]. However, the first cycle coulombic efficiency was only 35%. The specific capacity of 1170 mAh/ g was obtained during the first charge (de-lithiation). The drastic capacity loss was observed in the subsequent cycles. After 10 cycles, the capacity dropped to lower than 200 mAh/g, which is insufficient for an anodes material. Much effort has been devoted to increasing the performance and cyclability of silicon materials. Synthesized nano-scaled morphology silicon and/or form silicon/carbon composite are among the most effective methods.

Nano-sized silicon demonstrated superior performance to micron sized silicon. Li *et al.* reported that decreasing the silicon particle size to the nanometer-scale (78 nm) can be achieved by laser-induced silane gas reaction [81]. By controlling the voltage range between 0 to 0.8 V, silicon nano powder anode showed a reversible capacity of 1700 mAh/g after 15 cycles. Due to the smaller volume variation by the distribution of nanoparticles, better capacity retention than normal Si powder (passed through 250 meshes) was observed. Cycling at a current density of 0.8 mA/cm^2^, the nano-silicon anode remained at a high capacity of 1500 mAh/g. Recently, Kim *et al.* reported that very small silicon nanoparticles were successfully prepared at 380 °C under high pressure [82]. It was found that the selection of surfactants is crucial for particle size control. By using various surfactants, 5, 10 and 20 nm-sized nanoparticles can be obtained. Cycling these materials between 0 and 1.5 V at a rate of 0.2 C, the capacity of 2500 mAh/g can be achieved for over 40 cycles.

#### 3.2.2. Silicon/Carbon Composite Anode

Usually, a Solid-Electrolyte Interphase (SEI) layer protects the anode from chemically reacting with the electrolyte; however, the silicon anode experiences dramatic volume changes on cycling, and the layer can be broken on expansion. Carbon coating or composite with silicon will also help to reduce the SEI film formation and irreversible capacity. Carbon has less surface area than nano-silicon, thus less electrode and electrolyte side reactions happen. Wang *et al.* prepared carbon containing nanosize silicon particles by mechanical milling. The first reversible capacity of silicon/carbon composite was 1039 mAh/g and a capacity of 794 mAh/g was maintained after 20 cycles [83].

Porous silicon carbon composite allows additional spaces for changes in volume, as well as fast transportation of Li-ion. Magasinski *et al.* produced particles of 15–30 micron Si-C porous composite from powders via the bottom-up assembly route [84]. Cycling this material at a C/20 rate, a reversible charge (deintercalation) capacity of about 1950 mAh/g can be reached. The specific capacity of the nanoparticles of Si alone was estimated to be 3670 mAh/g, which is close to the theoretical value. Under a current rate of 1C, the specific capacity of the Sn-C porous composite was stable up to 100 cycles at about 1500 mAh/g. The carbon nano networks in porous silicon carbon composite serve as both an efficient conducting network for Li-ion and electrons and as an elastic buffer with sufficient internal porosity to accommodate the silicon volume expansion.

Graphene sheets can also act as matrices for silicon and buffer changes in a nanoparticle volume well. Lee *et al.* fabricated well-dispersed Si nanoparticles supported by a 3D network of graphene sheets [85]. The Si/graphene composites exhibit high lithium ion storage capacity and cycling stability (>1500 mAh/g after 200 cycles). Intimate contact between nanoparticles and graphene sheets are essential for improved electrochemical performance.

**Figure 10:**
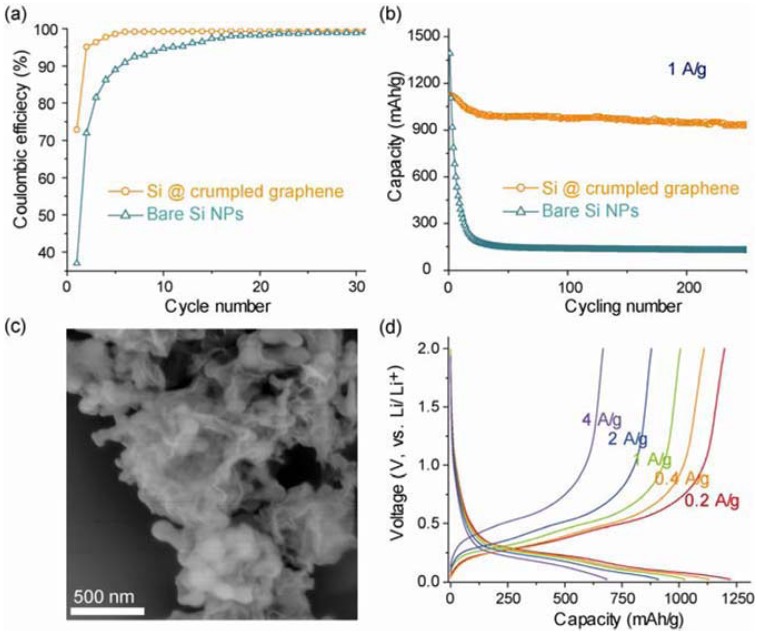
Electrochemical performance of the Si–crumpled graphene. (**a**) Coulombic efficiency and (**b**) charge/discharge cycling test of the composite capsules in comparison to the unwrapped Si nanoparticles at a constant current density of 1 A/g. (**c**) SEM image of the capsules after 250 cycles. (**d**) Charge/discharge profiles of the composite s electrode at various current densities ranging from 0.2 to 4 A/g. Adapted with permission from [86]. Copyright 2012 American Chemical Society.

Luo *et al.* developed a one-step aerosol assisted capillary assembly technique to create crumpled capsules of graphene-wrapped Si nanoparticles. As shown in Figure 10, the composite delivered about 1200 mAh/g at a low current density of 200 mA/g. When the current density was increased 20 times from 0.2 to 4 A/g, half the capacity (600 mAh/g) still remained [86].

#### 3.2.3. New Binder Improved Silicon Performance

Another way to improve the lifetime of silicon-based anodes is to optimize the binders, which keep the particles together causing them to adhere to the current collector during electrochemical cycling. Thermoplastic materials with a poor elastomeric property, such as polyvinylidene fluoride (PVDF), have been widely used as conventional binders for graphite and alloy anodes. However, PVDF may not be able to accommodate Si volume expansion on Li insertion. Kovalenko *et al.* demonstrated that alginate, a natural polysaccharide extracted from brown algae, shows much better properties to help silicon anodes achieve excellent performance compared to carboxymethyl cellulose (CMC) and PVDF [87].They proposed that the ideal binder for silicon should have a weak binder-electrolyte interaction, easy access to the silicon and the ability for building stable SEI film. Those are properties that are very necessary for long-term silicon anode cycling stability. Alginate appears to combine all of the above advantages. Therefore, it significantly improved the electrochemical performance of the silicon anode. At a current density of 4200 mA/g, the reversible Li extraction-specific capacity of an alginate-based Si anode is in the range of 1700 to 2000 mAh/g. In order to reduce the volume change during each cycling, a maximum of 1200 mAh/g capacity of Li insertion of each cycle was applied (about 30% to 40% depth of discharge). Thus the silicon anode shows remarkably stable anode performance for more than 1300 cycles. Coulombic efficiency approaching 99.9% was observed during cycling of the alginate-based Si anode, thus the alginate binder might also help to contribute in building a stable passivating SEI layer.

## 4. Current & Future Developments

Significant progress in lithium-ion battery technology has been made in the past decade. However, so far, long-term performance targets for all-electric vehicles have not been met. For the anodes, although lithium has a very high specific capacity (3860 mAh/g), dendrite formation and the nature of its high reactivity prevents its use in the Li-ion cells. Tin and silicon-based materials have great potential to replace graphite, because both have high gravimetric energy density and volumetric energy density. Advanced nanostructure Tin-based anodes have been developed with a cycle life of over 200 cycles and a reversible capacity above 500 mAh/g. The high capacity and stable cycle life has been achieved by decreasing particle size, amorphization of particles to enhance tolerance, accommodation by hollow structure or making composites with carbon and/or inactive components as buffering. Unfortunately, all these strategies have some kind of a disadvantage in reducing the overall energy density of anodes (e.g., added carbon constituted over half the weight of the composite, large void in the hollow structure and low tap density of nanoparticulated materials). Recently, the major challenge of these materials has been partially overcome by the smart designed micron-sized particles (aggregated nano-particles). The great improvements in Li-ion batteries are also expected from cathodes, since the gravimetric capacity of the best cathode is less than one third of tin-based anodes. So far, there are no cathode materials, which have achieved more than 250 mAh/g with a high potential and excellent cycling.

Beyond lithium ion technology, the rechargeable magnesium (Mg) battery system is one of the most promising candidates, because it has higher volumetric capacity and a potentially lower cost (3833 mAh/cm^3^ and $2700/ton, respectively, for metal Mg anode). The theoretical volumetric energy density of a Mg/S battery exceeds 4500 Wh/L. This is almost twice that of a Li-ion battery composed of a graphite anode and a lithium cobalt dioxide cathode [88]. Recently, Toyota demonstrated proof of the concept for the first rechargeable Mg/S battery [89]. Although more efforts will be needed to ameliorate the dissolution of sulfur and polysulfides, the specific capacity of 1200 mAh/g in the first cycle is quite encouraging. A new class of electrolytes based on Mg(BH_4_)_2_ for a Mg battery was also developed [90]. The performance of the cathode active material for the Mg battery is a critical issue to improve the energy and power density of the Mg battery. By using Mg metal as the anode, *α*-MnO_2_ exhibited a high reversible capacity of about 240 mAh/g during the first cycle [91]. Besides the pure Mg metal anodes, alternative insertion-type anodes, such as bismuth and tin, have been developed [92,93]. These anodes offer the opportunity for the potential use of conventional battery electrolytes in the magnesium-ion batteries.

The development and commercialization of lithium ion batteries is rooted in material discovery and reduction of the manufacturing cost. In the near future, achieving the goal toward alternative forms of transportation, such as plug-in hybrid electric vehicles (PHEV) and all electric vehicles (EV), relies on either the discovery of novel Li-ion battery materials, which have very high energy density, or the novel multi-electron transfer system.
